# Neoadjuvant gemcitabine and carboplatin followed by immediate cystectomy may be associated with a survival benefit in patients with clinical T2 bladder cancer

**DOI:** 10.1007/s12032-014-0949-9

**Published:** 2014-04-04

**Authors:** Takuya Koie, Chikara Ohyama, Hayato Yamamoto, Atsushi Imai, Shingo Hatakeyama, Takahiro Yoneyama, Yasuhiro Hashimoto, Tohru Yoneyama, Yuki Tobisawa

**Affiliations:** Department of Urology, Hirosaki University Graduate School of Medicine, 5 Zaifucho, Hirosaki, 036-8562 Japan

**Keywords:** Muscle-invasive bladder cancer, Neoadjuvant chemotherapy, Cystectomy, Clinical T2

## Abstract

Neoadjuvant cisplatin-based chemotherapy for muscle-invasive bladder cancer (MIBC) is more beneficial for clinical T3/4 than clinical T2 (cT2) disease. The aim of this study was to assess whether neoadjuvant GCarbo has a survival impact on cT2 bladder cancer. We retrospectively reviewed the medical records of 363 consecutive patients who underwent radical cystectomy (RC) between April 1997 and May 2012. We focused on 150 patients with cT2 MIBC. Seventy-nine patients received neoadjuvant GCarbo between March 2005 and April 2013. These patients received two courses of GCarbo and RC, and bilateral pelvic lymph node dissection (PLND) was performed at an interval of 1 month after chemotherapy. The control cohort included 71 patients with cT2 bladder cancer treated with RC and bilateral PLND alone between May 1994 and May 2007. Propensity score matching was used to adjust for potential selection biases associated with the treatment types. The endpoints were overall (OS), disease-specific (DSS), and disease-free survival (DFS). Propensity score-matched analysis resulted in 71 matched pairs from both groups. The 5-year OS rate was 98.6 % for the neoadjuvant GCarbo group and 66.6 % for the RC-alone group (*p* < 0.0001). The 5-year DSS rate was 100 % for the neoadjuvant GCarbo group and 69.7 % for the RC-alone group (*p* < 0.0001). The 5-year DFS rate was 94.2 % for the neoadjuvant GCarbo group and 72.7 % for the RC-alone group (*p* < 0.0001). In cT2 MIBC patients, neoadjuvant GCarbo chemotherapy followed by immediate cystectomy may improve OS and DFS compared to RC alone.

## Introduction

Radical cystectomy (RC) remains the gold standard treatment for muscle-invasive bladder cancer (MIBC). Although the surgical technique with RC and perioperative care has improved in recent years, approximately 50 % of MIBC patients will develop distant metastasis and eventually die of bladder cancer [1–3]. Similarly, despite local definitive therapy, the average 5-year disease-specific survival (DSS) rate for patients with MIBC is reported to be only around 50 % [4]. Hence, effective systemic therapy is necessary to achieve better oncological outcomes.

There is increasing evidence supporting the use of neoadjuvant chemotherapy (NAC) in locally advanced bladder cancer (BC). A meta-analysis of randomised trials showed that cisplatin-based NAC improves overall survival (OS) by 5 % in T2–T4a BC patients [5, 6]. However, despite these data, the use of perioperative chemotherapy for BC, in particular in the neoadjuvant setting, remains limited.

Although comparative trials on different NAC regimens are lacking, cisplatin-based combinations remain the most commonly used, because of their efficacy in selected patients and their relatively well-tolerated toxicity profile [7]. However, side effects associated with cisplatin-based regimens can be problematic, and approximately 40 % of patients are ineligible for cisplatin-based chemotherapy [8]. A review of the perioperative chemotherapy regimens used in patients with locally advanced BC in the USA between 1998 and 2003 showed that 10.4 and 1.2 % of patients received adjuvant chemotherapy and neoadjuvant chemotherapy, respectively [9]. Therefore, in this study, we adopted neoadjuvant gemcitabine and carboplatin (GCarbo) chemotherapy in patients with MIBC, instead of cisplatin [10].

Neoadjuvant chemotherapy (NAC) has several advantages: it may help in downstaging the BC before RC and compliance and tolerability of the chemotherapy regimen are better when given before surgery. The most important rationale for using NAC is to eradicate any potential micrometastases before RC and to avoid local/distant failure. NAC treatment has been demonstrated to improve survival inpatients with clinical T3/4 disease, compared to inpatients with clinical T2 (cT2) disease, and most of the available data suggest that the survival benefits of NAC are modest for cT2 MIBC patients [11]. However, NAC with methotrexate, vinblastine, doxorubicin, and cisplatin (MVAC), as validated by the Southwest Oncology Group (SWOG) 8710 trial, showed a survival advantage of 105 versus 75 months for NAC plus RC versus RC alone, in patients with cT2 BC [12]. Based on the conflicting results of these previous studies, we here aimed to assess whether neoadjuvant GCarbo has a survival impact on cT2 bladder cancer, using propensity score-matched analysis.

## Patients and methods

### Study population

In this retrospective study, we reviewed the clinical and pathological records of 327 consecutive MIBC patients who underwent RC and bilateral pelvic lymphadenectomy (PLND) with or without NAC between May 1994 and May 2013 at Hirosaki University, Japan. We focused on 147 patients with cT2 MIBC, including 79 patients who received neoadjuvant GCarbo (NAC group) and 71 patients who underwent RC and bilateral PLND only (RC-alone group). Eligible patients had histologically confirmed cT2 MIBC without distant metastasis. Information regarding patient demographics and tumour characteristics was obtained from the patients’ medical charts. At our institution, all MIBC patients received neoadjuvant GCarbo therapy beginning in 2005.

The study protocol and informed consent documents were reviewed and approved by the Hirosaki University institutional review board.

### Treatment schedule

All treatments were carried out at our institution. The patients who were treated before 2004 underwent standard surgical procedure using transperitoneal approach [13]. Patients in the NAC group received two cycles of GCarbo (800 mg/m^2^ of gemcitabine on days 1, 8, and 15 and carboplatin at an area under the curve (AUC) of 4 according to Calvert’s formula [14] on day 2) as neoadjuvant chemotherapy. Each cycle lasted for 21 days. The two courses of neoadjuvant chemotherapy were followed by RC and PLND at an interval of 1 month [15]. In NAC group, RC was performed via a 7-cm midline incision and the urinary bladder was removed along the non-incised peritoneum [15]. The choice of urinary diversion was determined according to the surgeon’s and/or patient’s preference. PLND, including the hypogastric, external iliac, obturator, presacral, and common iliac lymph nodes up to the aortic bifurcation, was routinely performed.

### Patient evaluation

Baseline evaluations included complete history taking and physical examinations, assessment of Eastern Cooperative Oncology Group performance status, abdominal and pelvic computed tomography (CT) or magnetic resonance imaging (MRI), and chest radiography or CT.

Tumours were measured at baseline and before RC. The response to treatment was assessed using the Response Evaluation Criteria in Solid Tumours, version 1.1 [16].

The diagnosis of MIBC was confirmed by a single pathologist at our institution after reviewing the results of transurethral resection (TUR) and the MRI performed at baseline.

We extensively examined the specimens obtained during cystoprostatectomy to identify the presence of BC. Pathological examination of complete transmural sections of the bladder wall was performed to accurately determine the pathological stage. Additionally, histological examination of several sections from various sites within the bladder, including the dome, anterior wall, lateral walls, posterior wall, trigone, and both ureters, was performed to identify superficial disease or a second primary tumour.

All tumours were staged according to the American Joint Committee on Cancer staging manual, 7th edition [17]. All lymph nodes from each designated site were submitted for examination, and representative sections of the surrounding fibroadipose tissue were also examined. The absence of cancer in the bladder and lymph node specimens was classified as pT0.

### Endpoints and statistical analysis

The primary endpoints were OS, DSS, and disease-free survival (DFS). The secondary endpoints were the overall response rate (ORR) and a classification of pT0 based on the cystectomy specimen.

Data were analysed using IBM SPSS Statistics 20 (IBM Corp., Armonk, NY, USA). Differences between the NAC and RC-alone groups were compared using the χ^2^ test for categorical variables and the Student’s *t* test or Wilcoxon rank-sum test for continuous variables. To reduce the effects of selection bias and potential confounders in this observational study, we developed a method to perform the propensity score analysis. Propensity scores were calculated for each patient using multivariate logistic regression, using the following covariates: age, gender, histopathology, clinical lymph node involvement, and tumour grade. This method accounts for imbalances in confounding factors among discrete study cohorts. Continuous and categorical factors were combined to yield a propensity score for each individual in the study population. Subsequently, individuals in each of the different study cohorts were matched to those in the reference cohort, according to their calculated propensity scores.

Survival after RC was examined using the Kaplan–Meier method, and survival in the subgroups was then analysed using the log-rank test. DFS was defined as the time from RC to the appearance of local or regional disease/metastasis or death. All *p* values were two-sided, and the significance level was set at a *p* value of <0.05.

## Results

### Patient characteristics

In the RC-alone group, 71 patients underwent RC and bilateral PLND between May 1994 and May 2007. In the NAC group, 79 MIBC patients received neoadjuvant GCarbo and underwent RC and bilateral PLND between March 2005 and April 2013. The median age of the patients was 70 years [interquartile rate (IQR), 63–75 years], and the median follow-up period was 60 months (IQR, 36–100 months). All patients were diagnosed with MIBC on the basis of histological examination of the TUR specimens.

Propensity score matching yielded 71 matched pairs of patients (shown in Table 1). In the matched cohorts, no significant difference was noted between the two groups for any covariate. All patients had performance statuses of 0, and none of the enrolled patients had cardiac disease or chronic heart failure at the time of surgery.

**Table 1:** Patient characteristics

Variable	NAC (*N* = 71)	RC alone (*N* = 71)	p*
Age (years), median (IQR)	68 (63–75)	70 (63–75)	0.7383
Gender, *n* (%)			
Male	58 (82)	53 (75)	0.3132
Female	13 (18)	18 (25)	
Tumour grade, *n* (%)			
2	24 (34)	17 (24)	0.1975
3	47 (66)	54 (76)	
Histopathology, *n* (%)			
UC	70 (99)	68 (96)	0.3138
AC	1 (1)	2 (3)	
SCC	0	1 (1)	
Estimated GFR, *n* (%)			
<60 mL/min/1.73 m^2^	32 (45)	21 (30)	0.0569
≥60 mL/min/1.73 m^2^	39 (55)	50 (70)	
Follow-up period (months), median, (IQR)	53 (37–74)	86 (32–132)	<0.001

*NAC* neoadjuvant chemotherapy, *RC* radical cystectomy, *IQR* interquartile range, *UC* urothelial carcinoma, *AC* adenocarcinoma, *SCC* squamous cell carcinoma, *GFR* glomerular filtration rate

* Calculated by the Student’s *t* test or the Wilcoxon rank-sum test

### Surgical outcomes

In the NAC group, all patients underwent RC and PLND within approximately 1 month after neoadjuvant GCarbo therapy. The median interval from the diagnosis of MIBC to RC was 62 days (IQR, 57–67 days). The median surgical time, including urinary diversion, was 270 min (IQR, 233–325 min) for the NAC group and 367 min (IQR, 271–445 min) for the RC-alone group (*p* < 0.001). The median estimated blood loss was 1,200 mL (IQR, 788–1,930 mL) in the NAC group and 1,250 mL (IQR, 836–2,194 mL) in the RC-alone group (*p* = 0.0878). The median lymph node count was 20 (IQR, 14–23) in the NAC group and 16 (IQR, 10–18) in the RC-alone group (*p* = 0.1856).

### Oncological outcomes

By the end of the follow-up period, 37 patients, including one patient in the NAC group and 36 in the RC-alone group, had died. In the RC-alone group, 20 patients died of BC and 16 died of other causes, including pneumonia in four patients, other cancers in four patients, cerebral haemorrhage in two patients, liver cirrhosis in one patient, and unknown causes in five patients. In the NAC group, no patient died due to BC, whereas three patients were alive with bladder cancer at the end of the follow-up period. The site of metastasis or recurrence was the lymph nodes in one patient and the right ureter in one patient, and one patient showed local recurrence.

**Fig. 1:**
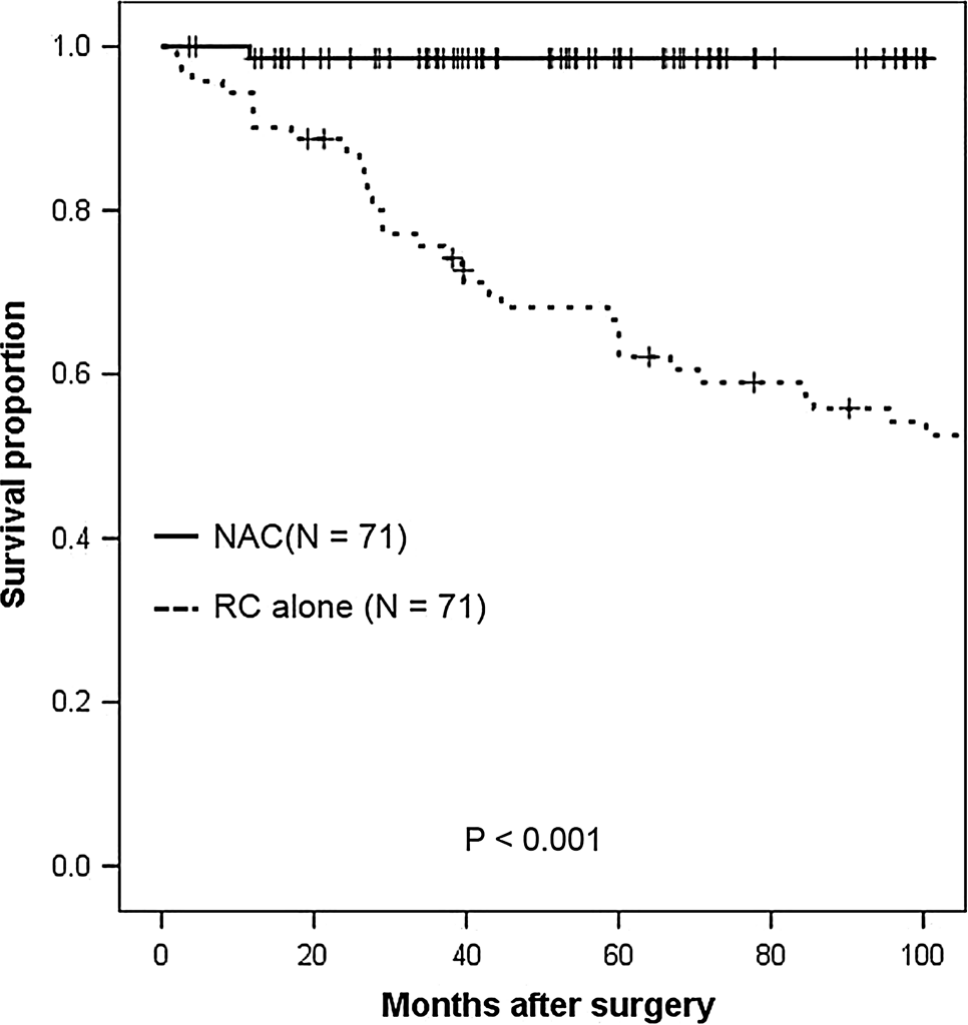
Kaplan–Meier analysis of overall survival in clinical T2 bladder cancer patients receiving radical cystectomy with or without neoadjuvant therapy. The 5-year overall survival rate was 98.6 % for patients who received neoadjuvant therapy followed by immediate cystectomy and 66.6 % for patients who underwent cystectomy alone (*p* < 0.001, log-rank test)

**Fig. 2:**
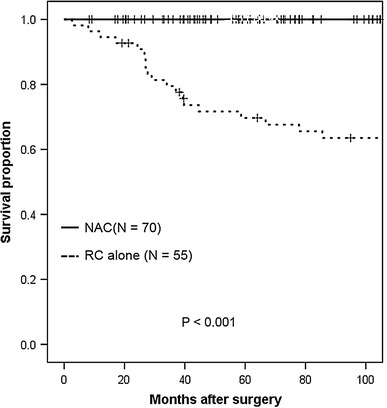
Kaplan–Meier analysis of disease-specific survival in clinical T2 bladder cancer patients who underwent radical cystectomy with or without neoadjuvant therapy. The 5-year disease-specific survival rates were 100 % for patients who received neoadjuvant therapy followed by immediate cystectomy, and 69.7 % for patients who underwent cystectomy alone (*p* < 0.001, log-rank test)

**Fig. 3:**
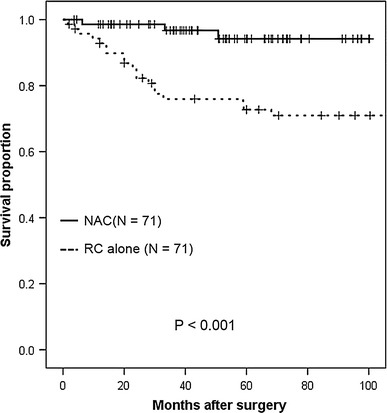
Kaplan–Meier analysis of disease-free survival in clinical T2 bladder cancer patients receiving radical cystectomy with or without neoadjuvant therapy. The 5-year disease-free survival rate was 94.2 % for patients who received neoadjuvant therapy followed by immediate cystectomy and 72.7 % for patients who underwent cystectomy alone (*p* < 0.001, log-rank test)

The 5-year OS rate was 98.6 % in the NAC group and 66.6 % in the RC-alone group (*p* < 0.001, log-rank test; shown in Fig. 1). The 5-year DSS rates were 100 % in the NAC group and 69.7 % in the RC-alone group (*p* < 0.001, log-rank test; shown in Fig. 2). The 5-year DFS rate was 94.2 % in the NAC group, compared to 72.7 % in the RC-alone group (*p* < 0.001, log-rank test; shown in Fig. 3).

### Clinical response in the NAC group

Twenty-two (31 %) of the 71 patients were not evaluable for clinical responses, because CT or MRI did not demonstrate any obvious, measurable tumours after diagnostic TUR. Out of the 59 patients who had measurable tumours, 13 (22 %) showed a complete response and 31 (53 %) showed a partial response, accounting for an ORR of 75 %. In addition, 13 patients (22 %) showed stable disease, and 2 (3 %) showed progressive disease.

### Pathological outcomes

All patients were evaluable for pathological outcomes. The pathological outcomes are shown in Table 2. Pathological staging based on the surgical specimens was pT0 in 22 (31 %) patients in the NAC group. Overall, eight patients (6 %) had pathologically identified lymph node involvement, and there was no significant difference between the two groups (*p* = 0.3622).

**Table 2:** Pathological distribution of patients with clinical T2 bladder cancer

Variable	NAC (*N* = 71)	RC alone (*N* = 71)
Pathological tumour stage, *n* (%)		
T0	22 (31)	0 (0)
T1	20 (28)	18 (25)
T2	24 (34)	42 (59)
T3	4 (6)	7 (10)
T4a	1 (1)	4 (6)
Lymph node status, *n* (%)		
Negative	68 (96)	66 (93)
Positive	3 (4)	5 (7)

*NAC* neoadjuvant chemotherapy, *RC* radical cystectomy

In the RC-alone group, 57 (80 %) patients had pathologically organ-confined disease (≤pT2N0) and 14 (20 %) patients were found to have extravesical disease or lymph node metastasis.

## Discussion

Muscle-invasive bladder cancer (MIBC) is a systematic disease, and metastases or local recurrence in patients undergoing RC are often caused by micrometastases at the time of surgery. Therefore, it is important to apply systemic therapy early, in order to eradicate the risk of micrometastases outside the surgical field. The most commonly referenced NAC trial is the SWOG 8710 trial, in which neoadjuvant MVAC followed by RC was compared to RC alone [12]. Although the difference in 5-year OS was not statistically significant (*p* = 0.06), the results of this study have been used as evidence of the superiority of NAC over RC alone. Many investigators have reported that the patients most likely to benefit from NAC are those with cT3/T4 disease or lymph node involvement and that the same is not true for patients with cT2 disease. Therefore, to assess whether neoadjuvant GCarbo chemotherapy has a clinical benefit in patients with cT2 MIBC, we conducted the current study using propensity score-matched analyses.

In this study, OS and DFS in cT2 BC patients who underwent neoadjuvant GCarbo followed by immediate RC were all significantly improved compared with those in patients who underwent RC alone. Unfortunately, previous data for the use of neoadjuvant chemotherapy in patients with cT2 BC are not as clear-cut. Niegisch et al. [7] recommended in their study that the current use of cisplatin-based NAC should be restricted to patients with undebatable non-organ-confined MIBC (cT3/T4) and/or lymph node-positive disease. In clinical ≤T2N0 patients, immediate cystectomy seems preferable because the treatment benefits of RC in these patients are questionable and a delay of RC may allow tumour progression [7]. Indeed, several authors have reported that a delay in RC results in worsening of the pathological stage and in diminished survival [18]. However, the SWOG 8710 trial demonstrated a 2.5-year survival advantage in patients with T2 disease who received neoadjuvant MVAC followed by RC (105 vs. 75 months, *p* = 0.05) [12]. In the current study, the interval from the diagnosis of MIBC to RC was as short as 62 days. If neoadjuvant GCarbo really does not have an effect on patients with MIBC, our regimen may still be advantageous in terms of avoiding a delay in RC. In addition, the timing of RC may be very important in achieving the optimal effects of neoadjuvant chemotherapy. Therefore, two courses of neoadjuvant GCarbo may be potentially advantageous for obtaining the maximum treatment effect with a combination of NAC followed by immediate RC.

Wide discrepancies exist between clinical staging and actual pathological staging in MIBC, with recent studies reporting that 43–73 % of patients with cT2 BC were upstaged on pathological assessment [19, 20]. Similarly, 52 % of the patients who underwent RC were understaged in another study [21]. Therefore, clinical staging of BC relies upon the evaluation of the TUR specimen, together with physical examination and cross-sectional imaging. CT may miss up to 40 % of lymph node metastases, and the accuracy of CT and MRI for staging has been reported to be 55 and 60 %, respectively [22, 23]. In the SWOG phase II study (S0219), 34 of 74 patients who received neoadjuvant cisplatin-based chemotherapy achieved cT0 status, as determined by CT and TUR specimen analysis [24]. Of these patients, 10 underwent immediate RC and 6 (60 %) of these patients had residual MIBC or lymph node metastases. In fact, the 5-year DSS rate was reported to be 73.5 % in patients with pathological T2N0 BC [2], as compared to 56.5 % in patients with clinical T2N0 BC [19]. We believe that difficulties in the accurate clinical staging of patients with MIBC, either before or after NAC, must be recognised.

The current study has several limitations. First, it was a retrospective study, with an inherent potential for bias. Second, the use of clinical staging might be associated with understaging or overstaging. Third, a relatively small number of patients were enrolled in this study, and the follow-up period was relatively short.

The oncological outcomes, including OS and DFS, were significantly improved in cT2 MIBC patients who received neoadjuvant GCarbo chemotherapy followed by immediate RC, as compared to inpatients who underwent RC alone. Hence, we suggest that the standard care for MIBC, especially for cT2 disease, may include NAC followed by immediate definitive surgery.

## References

[1] Manoharan M, Katkoori D, Kishore TA, Kava B, Singal R, Soloway MS (2009). Outcome after radical cystectomy in patients with clinical T2 bladder cancer in whom neoadjuvant chemotherapy has failed. BJU Int.

[2] Hautman RE, de Petriconi RC, Pfeiffer C, Volkmer BG (2012). Radical cystectomy for urothelial carcinoma of the bladder without neoadjuvant or adjuvant therapy: long-term results in 1100 patients. Eur Urol.

[3] Gore JL, Litwin MS, Lai J, Yano EM, Madison R, Setodji C (2010). Use of radical cystectomy for patients with invasive bladder cancer. J Natl Cancer Inst.

[4] de Vries RR, Nieuwenhuijzen JA, Vincent A, van Tinteren H, Horenblas S (2010). Survival after cystectomy for invasive bladder cancer. Eur J Surg Oncol.

[5] Winquist E, Kirchner TS, Segal R, Chin J, Lukka H (2004). Neoadjuvant chemotherapy for transitional cell carcinoma of the bladder: a systematic review and meta-analysis. J Urol.

[6] Herr HW, Dotan Z, Donat SM, Bajorin DF (2007). Defining optimal therapy for muscle invasive bladder cancer. J Urol.

[7] Niegisch G, Lorch A, Droller MJ, Lavery HJ, Stensland KD, Albers P (2013). Neoadjuvant chemotherapy in patients with muscle-invasive bladder cancer: which patients benefit?. Eur Urol.

[8] Canter D, Viterbo R, Kutikov A, Wong YN, Plimack E, Zhu F (2011). Baseline renal function status limits patient eligibility to receive perioperative chemotherapy for invasive bladder cancer and is minimally affected by radical cystectomy. Urology.

[9] David KA, Milowsky MI, Ritchey J, Carroll PR, Nanus DM (2007). Low incidence of perioperative chemotherapy for stage III bladder cancer 1998–2003: a report from the National Cancer Data Base. J Urol.

[10] Koie T, Ohyama C, Hashimoto Y, Hatakeyama S, Yamamoto H, Yoneyama T (2013). Efficacies and safety of neoadjuvant gemcitabine plus carboplatin followed by immediate cystectomy in patients with muscle-invasive bladder cancer, including those unfit for cisplatin: a prospective single-arm study. Int J Clin Oncol.

[11] Calabro F, Sternberg CN (2009). Neoadjuvant and adjuvant chemotherapy in muscle-invasive bladder cancer. BJU Int.

[12] Grossman HB, Natale RB, Tangen CM, Speights VO, Vogelzang NJ, Trump DL (2003). Neoadjuvant chemotherapy plus cystectomy compared with cystectomy alone for locally advanced bladder cancer. N Eng J Med.

[13] Stein JP, Skinner DG (2004). Surgical atlas. Radical cystectomy. BJU Int.

[14] Calvert AH, Newell DR, Gumbrell LA, O’Reilly S, Burnell M, Boxall FE (1989). Carboplatin dosage: prospective evaluation of a simple formula based on renal function. J Clin Oncol.

[15] Koie T, Ohyama C, Yamamoto H, Hatakeyama S, Kudoh S, Yoneyama T (2012). Minimum incision endoscopic radical cystectomy in patients with malignant tumors of the urinary bladder: clinical and oncological outcomes at a single institution. Eur J Surg Oncol.

[16] Eisenhauer EA, Therasse P, Bogaerts J, Schwartz LH, Sargent D, Ford R (2009). New response evaluation criteria in solid tumours: revised RECIST guideline (version 1.1). Eur J Cancer.

[17] Chang SS, McKiernan JM, Amin M, Bochner BH, Campbell S, Gospodarowics MK, et al. Urinary bladder. In: Edge SB, Byrd DR, Compton CC, et al., editors. AJCC Cancer Staging Manual, 7th edition. New York: Springer; 2010, pp. 497–505.

[18] Weight CJ, Garcia JA, Hansel DE, Fergany AF, Campbell SC, Gong MC (2009). Lack of pathologic down-staging with neoadjuvant chemotherapy for muscle-invasive urothelial carcinoma of the bladder. A contemporary series. Cancer.

[19] Canter D, Long C, Kutikov A, Plimack E, Saad I, Oblaczynski M (2011). Clinicopathological outcomes after radical cystectomy for clinical T2 urothelial carcinoma: further evidence to support the use of neoadjuvant chemotherapy. BJU Int.

[20] Hollenbeck BK, Miller DC, Dunn RL, Montie JE, Wei JT (2005). The effects of stage divergence on survival after radical cystectomy for urothelial cancer. Urol Oncol.

[21] Cheng L, Neumann RM, Weaver AL, Cheville JC, Leibovich BC, Ramnani DM (2000). Grading and staging of bladder carcinoma in transurethral resection specimens. Correlation with 105 matched cystectomy specimens. Am J Clin Pathol.

[22] Paik ML, Scolieri MJ, Brown SL, Spirnak JP, Resnick MI (2000). Limitations of computed tomography in staging invasive bladder cancer before radical cystectomy. J Urol.

[23] Buy JN, Moss AA, Guinet C, Ghossain MA, Malbec L, Arrive L (1988). MR staging of bladder carcinoma: correlation with pathologic findings. Radiology.

[24] de Vere White RW, Lara PN, Goldman B, Tangen CM, Smith DC, Wood DP (2009). A sequential treatment approach to myoinvasive urothelial cancer: a phase II South Oncology Group trial (S0219). J Urol.

